# Normative Data for Handgrip Strength in Saudi Older Adults Visiting Primary Health Care Centers

**DOI:** 10.3390/medicina55060251

**Published:** 2019-06-06

**Authors:** Saad M. Bindawas, Vishal Vennu, Saada M. Al-Orf, Sulaiman A. Alshammari, Maysoon M. Al-Amoud, Philip C. Calder, May N. Al-Muammar, Adel A. Alhamdan

**Affiliations:** 1 Department of Rehabilitation Sciences, College of Applied Medical Sciences, 10219 King Saud University, Riyadh 11433, Saudi Arabia; vvennu@ksu.edu.sa; 2 Department of Community Health Sciences, College of Applied Medical Sciences, King Saud University, Riyadh 11433, Saudi Arabia; smalorf@ksu.edu.sa (S.M.A.-O.); malmuammar@ksu.edu.sa (M.N.A.-M.); adel@ksu.edu.sa (A.A.A.); 3 Department of Family and Community Medicine, College of Medicine, 2925 King Saud University, Riyadh 11461, Saudi Arabia; sulaiman@ksu.edu.sa; 4 General Directorate of Primary Health Care Centers, Ministry of Health, Riyadh 11176, Saudi Arabia; drmamoud@gmail.com; 5 Human Development and Health, Faculty of Medicine, University of Southampton, Southampton, UK; NIHR Biomedical Research Centre, University Hospital Southampton NHS Foundation Trust and University of Southampton, Southampton SO17 1BJ, UK; pcc@soton.ac.uk

**Keywords:** normative data, hand grip strength, Saudi adults, cross-sectional study, primary health care

## Abstract

*Background and objective*: Handgrip strength (HGS) plays a vital role as a predictor of adverse health outcomes. Several studies have established HGS norms by age, sex, hand, occupation, culture or disability in different countries and for children in Saudi Arabia. However, standardized values for Saudi older adults have not yet been reported. Therefore, the current study was aimed to establish normative data for HGS in Saudi older adults visiting primary health care centers (PHCCs). *Material and Method*: In this descriptive cross-sectional study, HGS in kilograms was measured using a hydraulic hand dynamometer in Saudi older adults (*n* = 2045) aged ≥60 years visiting 15 PHCCs selected randomly from the five geographical regions of Riyadh, Saudi Arabia between January 2015 and April 2017. The average mean from three successive trials, standard deviations, and 95% confidence intervals presented for the left and right hands of men and women in six age groups (60–64, 65–69, 70–74, 75–79, 80–84, and 85+ years). The analyses were performed using the ANOVA test for all the age groups and to determine whether any differences exist between them. *Results*: The average mean HGS was significantly (*p* < 0.0001) differ by the left and right hands of men and women in six age groups. *Conclusions*: The current study presents specific norms for HGS in Saudi older adults by age, sex, and hand. Further studies are required to examine the utility of these norms for prediction of morbidity and mortality in this population.

## 1. Introduction

One of the most significant critical functions of the hand is grip strength, which can be used to study overall muscular strength [1]. Handgrip strength (HGS) plays a vital role as a predictor of disability [2], disorders (such as frailty and sarcopenia) [3], and adverse health outcomes, including mortality and hospital discharge [4]. Accordingly, HGS strength has been widely used to assess sarcopenia because it correlates well with lower extremity muscle strength [5]. This muscle strength is relevant to gait and physical and neural functions and how these are altered during aging [5]. These functions can be evaluated by using the Time Up and Go test (TUG), which is simple to conduct and correlates well with the HGS [6]. However, low HGS is a single test to assess manifestations of frailty, loss of functional independence and reduced quality of life although these are multifactorial [5]. It has been reported that HGS is associated with an increased risk of mortality from all causes, including cardiovascular disease and cancer [7]. It has also been found to be associated with demographics, such as age [8], sex [9], body construct (height, weight, bone mineral density, hand size, upper arm circumference, hand dominance) [10,11], and socioeconomic status (occupation, social status, and lifestyle) [11]. Moreover, it has been reported previously that HGS significantly differs between ethnicities [12,13].

At present, HGS is measured for healthy people in many countries, and its norms by age and sex are used in clinical practice [14]. Indeed, a meta-analysis of normative data showed that HGS varies depending on age and sex [15] and therefore, standard reference values by category are essential to make informed decisions about the normality of an individual’s status [16] and to set treatment goals and evaluate outcomes [17].

Several studies have established normative values for HGS in different countries [16,18,19], and indicated a significant variation in the norms by age group, sex, occupation, culture or disability. In general, men have greater HGS than women, and a gradual decline with increasing age has been reported [14]. Furthermore, HGS was found to be higher in the right than the left hand in both sexes [20] and was significantly lower in the population of developing than developed countries [15].

The studies mentioned above suggest that a universal standard for HGS norms does not exist. Owing to the different culture, lifestyles, and occupations [21] and the predominance of older people in Saudi Arabia [22], it is mandatory that HGS norms be established for the overall wellbeing and to design better treatment strategies. Recent research has shown that age is one of the predictors of HGS in healthy Saudi adult men [23].

Normative data of HGS has been reported for children aged 6–12 years in Saudi Arabia [24]. To the best of our knowledge, standardized values for older adults has not been reported yet. Therefore, the current descriptive study aimed to establish normative data for HGS in Saudi older adults visiting primary health care centers (PHCCs).

## 2. Materials and Methods

### 2.1. Study Design

The study was a descriptive cross-sectional study with a multistage stratified sampling strategy carried out in PHCCs, regulated by the Ministry of Health (MOH) in Riyadh, Saudi Arabia between January 2015 and April 2017.

### 2.2. Participants

This study included data from 2045 adults visiting 15 PHCCs selected randomly from the five geographical regions of Riyadh. Three PHCCs has chosen from each of the following five areas: north, south, central, east, and west. Men and women aged ≥60 years attending the selected PHCCs for routine primary care services were included. Before conducting the investigation, the researchers received approval from the MOH. All participants provided their informed consent before enrolling. 

This study is approved by the primary care and preventive medicine administration at the Ministry of Health, Saudi Arabia (reference: 10S/72) on 16 September 2012.

### 2.3. Measures

We assessed the HGS using the Jamar^®^ Hydraulic Hand Dynamometer (Patterson Medical [formerly Sammons Preston], Warrenville, IL, USA) setting at the second handle position using standard procedures [25]. A healthcare provider gave verbal instructions and demonstrated each testing procedure before assessments were performed. The scores were recorded for three successive trials for each hand tested. The average rating in kilograms (kg) for the right and left hand of each participant was calculated from the highest scores of two consecutive trials. The average mean score was calculated to quantify the amount of static force that the hand can squeeze around a dynamometer. If participants did not maintain the correct position during testing, the assessor discarded the measurement and repeated the test to produce accurate and acceptable HGS measures [26].

A trained health care provider obtained each participant’s socio-demographic variables including age, marital status, educational status, and occupational status along with health data using a structured questionnaire. Body Mass Index (BMI) was determined by dividing the weight in kg by the square of height in meters. A self-reported questionnaire was used to assess each participant’s activity of daily living, such as eating, bathing, dressing, and toileting; the response was recorded according to the degree of difficulty (none, a little, a lot, unable to). A score of 6 represents being fully independent and 2 or less indicates dependence [27].

Results of TUG test were recorded wherein each participant was asked to rise from an armchair (seat height 49 cm), walk 3 m, turn around (180 degrees), walk back and sit back down in the chair [28]. The time was recorded in seconds using a stopwatch. One practice trial was performed, and assistive devices were allowed.

### 2.4. Statistical Analyses

Characteristics of participants are reported as the mean ± standard deviation (SD) for continuous variables and count (percentage) for categorical variables. An independent student t-test for mean values and Chi-square test for frequencies were used to determine the significant difference between the sexes. The average mean from three successive trials, standard deviations (SD), and 95% confidence intervals (CI) presented for the left and right hands of men and women in six age groups (60–64, 65–69, 70–74, 75–79, 80–84, and 85+ years). The analyses were performed using the ANOVA test for all the age groups and to determine whether any differences exist between them. All analyses were performed using SPSS, version 22.0 (SPSS Inc., Chicago, IL, USA) for Windows^®^. A *p*-value of <0.05 was considered statistically significant.

## 3. Results

### 3.1. Participant’s Characteristics

The descriptive statistics for all the study participants are presented in Table 1. A total of 2045 participants (1138 men, and 907 women) were included in this study. The average age of the participants was 66 years, and dominant sex was men (55.6%). Both sexes were significantly (*p* < 0.001) differ by age, age groups, marital status, education, occupation, and BMI.

### 3.2. Results Related to Normative Data

The average TUG score was significantly (*p* < 0.001) higher in women (16.6 ± 9.5 s) compared to men (12.2 ± 6.5 s). Most of the participants (91%) were right-handed. Normative data of HGS for the right and left hands of men and women in six age groups are presented in Table 2.

### 3.3. Comparison between the Present Study and Other Countries Norms 

**Figure 1:**
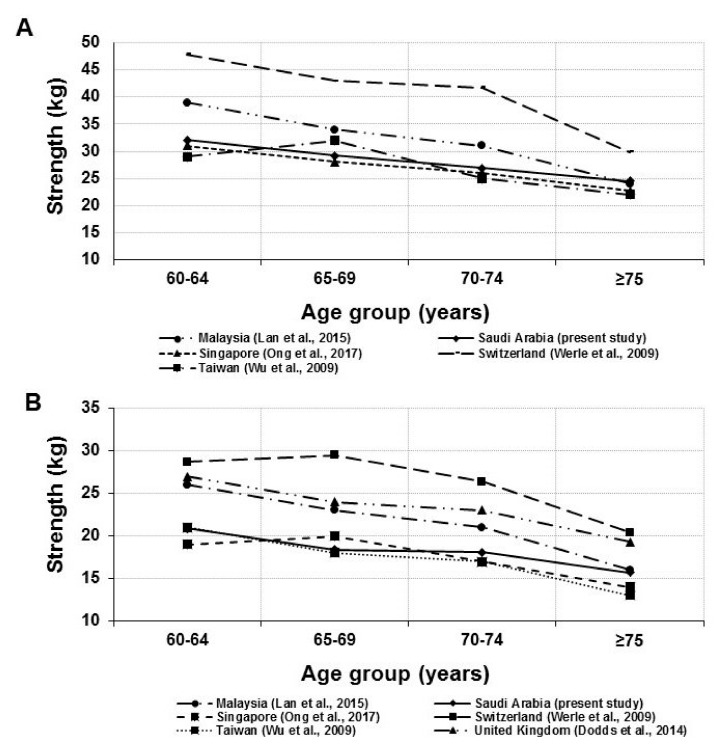
Mean handgrip strength of the right hand (kg) of older (**A**) men and (**B**) women over four successive age ranges (from 60 years) in Saudi adults compared with other countries norms.

The mean HGS norms of the right hand of older Saudi men and women over four successive age ranges from 60 years compared to other countries are illustrated in Figure 1. As shown in the figure, the mean HGS decreased with increasing age in all countries. The mean HGS values among Saudi older men and women visiting PHCCs are close to Singapore [29], and Taiwan norms [30].

## 4. Discussion

The establishment of normative data for HGS plays a crucial role in disease prediction for many morbidities [4] and mortality from all causes [7]. Therefore, the current study was aimed to establish normative data for HGS in Saudi older adults visiting PHCCs. Our study results indicate that the average mean HGS varied significantly by the left and right hands of men and women in six age groups.

Our study findings broadly support the results of studies conducted by others [14,19,31,32] with ample justification for separate reference values by age and sex. However, the current study population differs from other studies [14,18,31,33,34,35] probably owing to differences in the recruitment of the study population along with variations in the categorization of subjects by age, sex, or geographical region. For example, an earlier study covered the normative data from the general population in Saudi Arabia, including adulthood (aged 50 years and above) and compared these with 27 countries in 7 United Nations regions [15]. Findings of that study revealed that average HGS are considerably lower in a developing country as Saudi Arabia compared with developed world regions. This finding highlighted that different cut points of HGS for the diverse population in various geographical areas may be needed.

An earlier study reported that the difference between the HGS scores of the right and left hands varies from 0% to 10% [36]; in the current study, the difference was of the order of 5 to 10% (depending upon age and sex). It is likely that handedness influences hand strength [31]. The present study findings are consistent with many previous studies which recruited subjects with demographics that are similar to our study [29,30,37].

Another main finding of this study was that both the right and left hands in women aged 60–64 years had less grip strength than seen in men of the same age group (about 11 kg less in both hands). This finding is generally similar to that of a previous study [19] which suggested that mean HGS for the right and left hands of the same age group women in a multiethnic Asian population differs by 13.5 kg and 12.7 kg, respectively than that of men. However, the difference between men and women seen in the current study was a little less than reported for a multiethnic Asian cohort of the same age [19] and a Korean cohort (14 kg less in women than men) aged 60–69 years [34]. These small differences might relate to physical factors, dietary factors and the overall well-being of modern society. Further studies are needed to confirm these variations. Clinicians should continue to consider using these normative values with regards to age, sex, and hand in their routine practice.

The current study is not devoid of limitations. Because of recruitment of subjects’ ≥60 years of age visiting PHCCs, the study findings may have limited generalizability across all age groups, including fully healthy populations. Moreover, the influence of participant factors, such as palm length, upper arm and waist circumferences [29] along with the participant’s hand sensations [38] may have influenced the results. However, estimation of HGS for the first time and establishment of normative values for the Saudi older populations is very useful for clinical practice assessments, patient’s follow-up and future research. Also, HGS was measured using a well-accepted hand dynamometer [26,39] and was found to be a reliable and valid tool to measure HGS in community-dwelling older adults [39]. Additionally, TUG was found to be accurate for measuring balance, functional mobility, and fall risk in older adults [40].

The findings from this study support important implications for the health care of older adults visiting PHCCs throughout Saudi Arabia. These HGS norms might allow clinicians to interpret or compare results by age, sex, and hand. These comparisons can help clinicians to gauge their patients HGS performance and decide to offer a standard treatment and intervention. Also, researchers could use these norms as a baseline to study the trend for comparison with future studies. Future studies are needed to accommodate participants from rural regions throughout Saudi Arabia.

## 5. Conclusions

The current study presents specific norms for HGS in Saudi older adults by age, sex, and hand. These population norms are close to the Singaporean and Taiwanese normative values. The study results are significant since they will help in the interpretation of HGS in clinical and research settings not only in Saudi Arabia but also in other Arab countries. Moreover, these norms play a crucial role in the estimation of standard performance as a basis for prescribing corrective interventions or predicting future performance. Further studies are required to examine the utility of these norms for prediction of morbidity and mortality in this population.

## Figures and Tables

**Table 1:** Study participants by sex (N = 2045).

Characteristics	Men	Women	*p*-Value
	(N = 1138)	(N = 907)	
Age in years	67.0 ± 6.8	65.0 ± 6.5	*p < 0.001*
Age group, (%)			
60–64 years	529 (46.5)	559 (61.6)	*p < 0.001*
65–69 years	238 (20.9)	149 (16.4)	
70–74 years	201 (17.7)	114 (12.6)	
75–79 years	97 (8.5)	44 (4.9)	
80-84 years	52 (4.6)	26 (2.9)	
>=85 years	21 (1.8)	15 (1.7)	
Marital status, (%)			
Married	1084 (95.3)	540 (59.5)	*p < 0.001*
Single	6 (0.5)	9 (1.0)	
Widow	45 (4.0)	304 (33.5)	
Divorced	3 (0.3)	54 (6.0)	
Education levels, (%)			
Illiterate	224 (19.7)	522 (57.6)	*p < 0.001*
Reads and write + Primary	337 (29.6)	214 (23.6)	
Intermediate + Secondary	416 (36.6)	137 (15.1)	
University or above	161 (14.1)	34 (3.7)	
Occupation, (%)			
Employed	259 (22.8)	74 (8.2)	*p < 0.001*
Not Employed	879 (77.2)	833 (91.8)	
BMI, kg/m^2^	28.5 ± 5.3	30.9 ± 5.7	*p < 0.001*
Underweight, (%)	18 (1.6)	7 (0.8)	
Normal, (%)	251 (22.8)	102 (12.0)	*p < 0.001*
Overweight, (%)	450 (40.9)	280 (32.8)	
Obese, (%)	380 (34.6)	464 (54.4)	
ADL, (%)			0.076
0–2	19 (1.7)	16 (1.8)	
3–5	115 (10.2)	121 (13.4)	
6	992 (88.1)	763 (84.8)	

SD, standard deviation; TUG, Time-Up and Go test, BMI, body mass index; ADL, the activity of daily living; kg, kilogram.

**Table 2:** Descriptive Statistics (Mean, SD, and 95% CI) for handgrip strength (kg), *n* = 2045.

Characteristics		Men (N = 1138)				Women (N = 907)			
		Right Hand		Left Hand		Right Hand		Left Hand	
		Mean ± SD	95% CI	Mean ± SD	95% CI	Mean ± SD	95% CI	Mean ± SD	95% CI
**60–64**	N = 1088	32.1 ± 7.2	31.5–32.6	30.5 ± 7.9	29.9–31.1	20.9 ± 7.4	20.2–21.5	19.3 ± 7.1	18.7–19.9
**65–69**	N = 387	29.2 ± 8.4	28.0–30.3	27.4 ± 7.5	26.4–28.4	18.4 ± 6.7	17.4–19.4	16.9 ± 6.6	15.9–17.9
**70–74**	N = 315	26.9 ± 7.2	25.8–27.9	25.1 ± 7.1	24.0–26.1	18.1 ± 7.0	16.9–19.2	17.0 ± 6.5	15.9–18.0
**75–79**	N = 141	25.8 ± 6.3	24.4–27.2	24.8 ± 6.1	23.4–26.1	17.0 ± 6.9	15.2–18.7	16.2 ± 6.6	14.5–17.8
**80–84**	N = 78	22.2 ± 7.4	20.0–24.4	20.2 ± 6.6	18.2–22.1	15.7 ± 6.4	13.6–17.7	15.0 ± 6.6	12.9–17.0
**>=85**	N = 36	25.4 ± 6.5	22.5–28.2	21.3 ± 5.8	18.7–23.8	14.4 ± 6.9	11.0–17.8	12.1 ± 5.4	9.4–14.7

SD, standard deviation; CI, confidence interval.

## References

[1] McGrath R.P., Kraemer W.J., Al Snih S., Peterson M.D. (2018). Handgrip Strength and Health in Aging Adults. Sports Med..

[2] Giampaoli S., Ferrucci L., Cecchi F., Lo Noce C., Poce A., Dima F., Santaquilani A., Vescio M.F., Menotti A. (1999). Hand-grip strength predicts incident disability in non-disabled older men. Age Ageing.

[3] Clegg A., Young J., Iliffe S., Rikkert M.O., Rockwood K. (2013). Frailty in elderly people. Lancet.

[4] Sasaki H., Kasagi F., Yamada M., Fujita S. (2007). Grip strength predicts cause-specific mortality in middle-aged and elderly persons. Am. J. Med..

[5] Carson R.G. (2018). Get a grip: Individual variations in grip strength are a marker of brain health. Neurobiol. Aging.

[6] Alonso A.C., Ribeiro S.M., Luna N.M.S., Peterson M.D., Bocalini D.S., Serra M.M., Brech G.C., Greve J.M.D., Garcez-Leme L.E. (2018). Association between handgrip strength, balance, and knee flexion/extension strength in older adults. PLoS ONE.

[7] Gale C.R., Martyn C.N., Cooper C., Sayer A.A. (2006). Grip strength, body composition, and mortality. Int. J. Epidemiol..

[8] Dodds R.M., Syddall H.E., Cooper R., Benzeval M., Deary I.J., Dennison E.M., Der G., Gale C.R., Inskip H.M., Jagger C. (2014). Grip strength across the life course: Normative data from twelve British studies. PLoS ONE.

[9] Oksuzyan A., Singh P.K., Christensen K., Jasilionis D. (2018). A Cross-National Study of the Gender Gap in Health Among Older Adults in India and China: Similarities and Disparities. Gerontologist.

[10] Hossain M.G., Zyroul R., Pereira B.P., Kamarul T. (2012). Multiple regression analysis of factors influencing dominant hand grip strength in an adult Malaysian population. J. Hand Surg. Eur. Vol..

[11] Bunout D., Barrera G., De La Maza T., Avendano M., Gattas V., Petermann M., Hirsch S. (2004). Lean and fat mass as determinants of muscle strength and insulin sensitivity in Chilean elderly subjects. J. Nutr. Health Aging.

[12] Rantanen T., Guralnik J.M., Leveille S., Izmirlian G., Hirsch R., Simonsick E., Ling S., Fried L.P. (1998). Racial differences in muscle strength in disabled older women. J. Gerontol. A Biol. Sci. Med. Sci..

[13] Haas S.A., Krueger P.M., Rohlfsen L. (2012). Race/ethnic and nativity disparities in later life physical performance: The role of health and socioeconomic status over the life course. J. Gerontol. B. Psychol. Sci. Soc. Sci..

[14] Günther C.M., Bürger A., Rickert M., Crispin A., Schulz C.U. (2008). Grip strength in healthy caucasian adults: reference values. J. Hand Surg. Am..

[15] Dodds R.M., Syddall H.E., Cooper R., Kuh D., Cooper C., Sayer A.A. (2016). Global variation in grip strength: A systematic review and meta-analysis of normative data. Age Ageing.

[16] Bohannon R.W., Peolsson A., Massy-Westropp N., Desrosiers J., Bear-Lehman J. (2006). Reference values for adult grip strength measured with a Jamar dynamometer: A descriptive meta-analysis. Physiotherapy.

[17] Bohannon R.W. (2008). Hand-grip dynamometry predicts future outcomes in aging adults. J. Geriatr. Phys. Ther..

[18] Leong D.P., Teo K.K., Rangarajan S., Kutty V.R., Lanas F., Hui C., Quanyong X., Zhenzhen Q., Jinhua T., Noorhassim I. (2016). Reference ranges of handgrip strength from 125,462 healthy adults in 21 countries: A prospective urban rural epidemiologic (PURE) study. J. Cachexia Sarcopenia Muscle.

[19] Lam N.W., Goh H.T., Kamaruzzaman S.B., Chin A.-V., Poi P.J.H., Tan M.P. (2016). Normative data for hand grip strength and key pinch strength, stratified by age and gender for a multiethnic Asian population. Singap. Med. J..

[20] Wong S.L. (2016). Grip strength reference values for Canadians aged 6 to 79: Canadian Health Measures Survey, 2007 to 2013. Health Rep..

[21] Bindawas S.M., Vennu V. (2018). The National and Regional Prevalence Rates of Disability, Type, of Disability and Severity in Saudi Arabia—Analysis of 2016 Demographic Survey Data. Int. J. Environ. Res. Public Health.

[22] Karlin N.J., Weil J., Felmban W. (2016). Aging in Saudi Arabia: An exploratory study of contemporary older persons’ views about daily life, health, and the experience of aging. Gerontol. Geriatr. Med..

[23] Alahmari K.A., Silvian S.P., Reddy R.S., Kakaraparthi V.N., Ahmad I., Alam M.M. (2017). Hand grip strength determination for healthy males in Saudi Arabia: A study of the relationship with age, body mass index, hand length and forearm circumference using a hand-held dynamometer. J. Int. Med. Res..

[24] Omar M.T.A., Alghadir A., Al Baker S. (2015). Norms for hand grip strength in children aged 6–12 years in Saudi Arabia. Dev. Neurorehabilit..

[25] Fiebert I.M., Roach K.E., Armstrong T., Mandel D.W., Donohue M. (1996). Dynamometric grip strength assessment of subjects sixty years and older. Phys. Occup. Ther. Geriatr..

[26] Wang C.-Y., Chen L.-Y. (2010). Grip strength in older adults: Test-retest reliability and cutoff for subjective weakness of using the hands in heavy tasks. Arch. Phys. Med. Rehabil..

[27] Wallace M., Shelkey M. (2007). Katz index of independence in activities of daily living (ADL). Urol. Nurs..

[28] Schoene D., Wu S.M.S., Mikolaizak A.S., Menant J.C., Smith S.T., Delbaere K., Lord S.R. (2013). Discriminative ability and predictive validity of the timed Up and Go test in identifying older people who fall: Systematic review and meta-analysis. J. Am. Geriatr. Soc..

[29] Ong H.L., Abdin E., Chua B.Y., Zhang Y., Seow E., Vaingankar J.A., Chong S.A., Subramaniam M. (2017). Hand-grip strength among older adults in Singapore: a comparison with international norms and associative factors. BMC Geriatr..

[30] Wu S.W., Wu S.F., Liang H.W., Wu Z.T., Huang S. (2009). Measuring factors affecting grip strength in a Taiwan Chinese population and a comparison with consolidated norms. Appl. Ergon..

[31] Werle S., Goldhahn J., Drerup S., Simmen B.R., Sprott H., Herren D. (2009). Age-and gender-specific normative data of grip and pinch strength in a healthy adult Swiss population. J. Hand Surg. Eur. Vol..

[32] Kamarul T., Ahmad T.S., Loh W. (2006). Hand grip strength in the adult Malaysian population. J. Orthop. Surg..

[33] McQuiddy V.A., Scheerer C.R., Lavalley R., McGrath T., Lin L. (2015). Normative values for grip and pinch strength for 6-to 19-year-olds. Arch. Phys. Med. Rehabil..

[34] Shim J.H., Roh S.Y., Kim J.S., Lee D.C., Ki S.H., Yang J.W., Jeon M.K., Lee S.M. (2013). Normative measurements of grip and pinch strengths of 21st century Korean population. Arch. Plast. Surg..

[35] Luna-Heredia E., Martin-Pena G., Ruiz-Galiana J. (2005). Handgrip dynamometry in healthy adults. Clin. Nutr..

[36] Beumer A., Lindau T.R. (2014). Grip strength ratio: A grip strength measurement that correlates well with DASH score in different hand/wrist conditions. BMC Musculoskelet. Disord..

[37] Malhotra R., Ang S., Allen J.C., Tan N.C., Østbye T., Saito Y., Chan A. (2016). Normative values of hand grip strength for elderly singaporeans aged 60 to 89 years: A cross-sectional study. J. Am. Med. Dir. Assoc..

[38] Fink B., Hamdaoui A., Wenig F., Neave N. (2010). Hand-grip strength and sensation seeking. Pers. Individ. Differ..

[39] Bohannon R.W., Schaubert K.L. (2005). Test–retest reliability of grip-strength measures obtained over a 12-week interval from community-dwelling elders. J. Hand Ther..

[40] Steffen T.M., Hacker T.A., Mollinger L. (2002). Age- and gender-related test performance in community-dwelling elderly people: Six-Minute Walk Test, Berg Balance Scale, Timed Up & Go Test, and gait speeds. Phys. Ther..

